# Supplementary material for: Risk Stratification for ECMO Requirement in COVID-19 ICU Patients Using Quantitative Imaging Features in CT Scans on Admission
Source: Diagnostics (Basel). 2021 Jun 3;11(6):1029. doi: 10.3390/diagnostics11061029 (PMC8228774; doi:10.3390/diagnostics11061029)
Supplement: Supplementary file 1 [file diagnostics-11-01029-s001.zip › diagnostics-1243826-SI.pdf]

Risk stratification for ECMO requirement in COVID-19 ICU patients using quantitative imaging features in CT scans on admission

E. Gresser[1], M.D., J. Reich[1], B. O. Sabel[1], M.D., W. G. Kunz[1], M.D., M. P. Fabritius[1], M.D., J. Rübenthaler, M.D., M. Ingrisch, PhD, Wassilowsky[2], M.D., M. Irlbeck[2], M.D., J. Ricke[1], M.D., D. Puhr-Westerheide[1], M.D.

[1]Department of Radiology, University Hospital, LMU Munich, Munich, Germany

[2]Department of Anesthesiology, University Hospital, LMU Munich, Munich, Germany

Supplemental Material

Corresponding author:

Eva Gresser, M.D.

Department of Radiology, University Hospital Munich, LMU Munich, Germany

Tel.: +49-89-4400-73620

Fax.: +49-89-4400-78832

Email: eva.gresser@med.uni-muenchen.de

Supplemental Table S1. Parameters associated with In-Hospital Mortality for COVID-19 ICU-patients

	In-Hospital Mortality		
Independent variables	Odds Ratio	CI	p value
Age	1.080	0.986-1.183	0.097
Sex	0.913	0.112-7.408	0.932
BMI	0.809	0.674-0.970	**0.022***
SOFA on Admission	0.886	0.667-1.178	0.406
Mean SOFA score	2.091	1.263-3.464	**0.004***
Maximum SOFA score	1.249	0.771-2.024	0.366
ECMO Therapy	5.811	0.452-74.744	0.177

Supplemental Table S1. Parameters associated with In-Hospital Mortality for COVID-19 ICU patients. Binary logistic regression analysis. ECMO, extracorporeal membrane oxygenation; COVID-19, coronavirus disease 2019; ICU, intensive care unit; CI, confidence interval; BMI, body mass index; SOFA, sequential organ failure assessment; CT, computed tomography; 8 patients not included in regression analysis due to missing values.

Supplemental Table S2. Predictors for ECMO Therapy for COVID-19 ICU-patients (VV-ECMO Patients only)

	ECMO Therapy		
Independent variables	Odds Ratio	CI	p value
Age	1.011	0.926-1.103	0.812
Sex	0.451	0.052-3.910	0.470
BMI	1.110	0.958-1.286	0.166
SOFA on Admission	1.309	1.054-1.626	**0.015***
Lactate on Admission	1.136	0.597-2.162	0.697
CT Lung Involvment (%) on Admission	1.053	1.006-1.101	**0.023***

Supplemental Table S2. Predictors for ECMO therapy (VV-ECMO patients only, n=12) for COVID-19 ICU patients. Results from binary logistic regression with adjustment for multiple covariates. ECMO, extracorporeal membrane oxygenation; COVID-19, coronavirus disease 2019; ICU, intensive care unit; CI, confidence interval; BMI, body mass index; SOFA, sequential organ failure assessment; CT, computed tomography; 18 patients not included in regression analysis due to missing values.

Table S3. Predictors for ECMO Therapy for COVID-19 ICU-patients

	ECMO Therapy		
Independent variables	Odds Ratio	CI	p value
Age	0.993	0.911-1.084	0.882
Hypertension	1.863	0.319-10.895	0.490
Diabetes	0.362	0.057-2.281	0.279
Renal Failure	9.467	0.600-149.356	0.110
Heart Disease	0.663	0.068-5.915	0.688
Pulmonary Disease	2.242	0.305-16.491	0.428
SOFA on Admission	1.376	1.080-1.754	**0.010***
CT Lung Involvment (%) on Admission	1.070	1.023-1,119	**0.003***

Supplemental Table S3. Comorbidity model for the prediction of ECMO therapy for COVID-19 ICU patients. Results from binary logistic regression with adjustment for multiple covariates. ECMO, extracorporeal membrane oxygenation; COVID-19, coronavirus disease 2019; ICU, intensive care unit; CI, confidence interval; SOFA, sequential organ failure assessment; CT, computed tomography; 18 patients not included in regression analysis due to missing values.
